# Drug repurposing using transcriptome sequencing and virtual drug screening in a patient with glioblastoma

**DOI:** 10.1007/s10637-020-01037-7

**Published:** 2020-12-12

**Authors:** Mohamed E. M. Saeed, Onat Kadioglu, Henry Johannes Greten, Adem Yildirim, Katharina Mayr, Frederik Wenz, Frank A. Giordano, Thomas Efferth

**Affiliations:** 1 Department of Pharmaceutical Biology, Institute of Pharmaceutical and Biomedical Sciences, Johannes Gutenberg University, Staudinger Weg 5, 5512 Mainz, Germany; 2 Heidelberg Clinics for Integrative Diagnostics, Heidelberg, Germany; 3 Cell and Matrix Biology, Institute of Zoology, Johannes Gutenberg University of Mainz, Mainz, Germany; 4 University Hospital Freiburg, Freiburg im Breisgau, Germany; 5 Department of Radiation Oncology, University Hospital Bonn, Bonn, Germany

**Keywords:** Drug repurposing, Precision medicine, Targeted chemotherapy, Virtual drug screening

## Abstract

*Background* Precision medicine and drug repurposing are attractive strategies, especially for tumors with worse prognosis. Glioblastoma is a highly malignant brain tumor with limited treatment options and short survival times. We identified novel BRAF (47-438del) and PIK3R1 (G376R) mutations in a glioblastoma patient by RNA-sequencing. *Methods* The protein expression of BRAF and PIK3R1 as well as the lack of EGFR expression as analyzed by immunohistochemistry corroborated RNA-sequencing data. The expression of additional markers (AKT, SRC, mTOR, NF-κB, Ki-67) emphasized the aggressiveness of the tumor. Then, we screened a chemical library of > 1500 FDA-approved drugs and > 25,000 novel compounds in the ZINC database to find established drugs targeting BRAF47-438del and PIK3R1-G376R mutated proteins. *Results* Several compounds (including anthracyclines) bound with higher affinities than the control drugs (sorafenib and vemurafenib for BRAF and PI-103 and LY-294,002 for PIK3R1). Subsequent cytotoxicity analyses showed that anthracyclines might be suitable drug candidates. Aclarubicin revealed higher cytotoxicity than both sorafenib and vemurafenib, whereas idarubicin and daunorubicin revealed higher cytotoxicity than LY-294,002. Liposomal formulations of anthracyclines may be suitable to cross the blood brain barrier. *Conclusions* In conclusion, we identified novel small molecules via a drug repurposing approach that could be effectively used for personalized glioblastoma therapy especially for patients carrying BRAF47-438del and PIK3R1-G376R mutations.

## Background

Half of the brain tumors represent diffuse gliomas, and the World Health Organization (WHO) provided classification criteria to predict the clinical behavior of these neoplasms [1]. Glioblastoma is classified as grade IV/IV gliomas with specific characteristics, including high cellularity, cellular pleomorphism, nuclear atypia and necrosis [2]. Glioblastoma has a dismal prognosis despite aggressive treatment [2, 3]. Various genetic mutations and aberration have been identified in glioblastoma patients, such as *MGMT* methylation [3, 4], BRAFG596A [5], BRAFV600E [5–7], PIK3R1G376R, PIK3R1D560Y, PIK3R1N564K mutations [8].

Mutations in proteins critical for cancer biology usually lead to uncontrolled cell growth, resistance to conventional chemotherapy and subsequently, therapy failure. In the past decade, the demand for effective novel agents to combat cancer has drawn the attention to specific molecular alterations in cancer cells as targets for therapy [9]. The concept of precision medicine implies the application of targeted drugs coupled with specific diagnostic assays in order to determine whether patients are likely to benefit from targeted therapy. The shift from classical, non-selective, cytotoxic chemotherapy to molecular targeted cancer drugs resulted in increased tumor response and patients’ survival rates [10–12].

Therapeutic monoclonal antibodies are considered a successful strategy for targeted cancer therapy. Antibodies have, however, several limitations, including targeting only cellular surface epitopes, high immunogenicity and high production costs [13]. Small molecule inhibitors possess advantages compared to antibodies, as they address both intracellular and surface proteins. A showcase example is the BCR-ABL inhibitor imatinib, which resulted in dramatic improvements in the survival of chronic myeloid leukemia patients. The same applies to small molecule inhibitors directed against targets in solid tumors, e.g. the epidermal growth factor receptor (EGFR) kinase inhibitors gefitinib and erlotinib to treat non-small cell lung cancer and the vascular endothelial growth factor receptor (VEGFR) kinase inhibitor sorafenib against renal cancer [14–16]. In the case of EGFR mutations in the kinase domain of the receptor, it happens that erlotinib is no longer therapeutically effective as cancer cells develop resistance to erlotinib. Numerous mutations appear during tumor progression and cause resistance [17, 18]. Therefore, it is essential to find novel inhibitors, which target and inhibit tumor-related mutant proteins.

The sequencing of tumor genomes and transcriptomes is more and more routinely applied in clinical oncology. The concept of precision medicine by mutations identified by sequence analyses may serve as a basis to select treatment options specifically addressing these mutations. Since such mutations would exclusively occur in tumors but not in normal tissues, it is expected that targeted treatments ought to provoke no or only minimal side effects. However, the vast majority of the data acquired cannot be used for therapeutic purposes yet, as we still lack drugs addressing all relevant mutations. Also, tumors frequently consist of heterogeneous subpopulations with mutational profiles different from the main cell population. Resistance to a targeted drug develop if subpopulations without the treatment-specific mutation appear. This may foster the fatal outcome of malignant diseases. Hence, one wishes to have a larger arsenal of drugs at hand to combat otherwise drug-resistant subpopulations of tumors with mutations, for which no targeted drugs are available currently.

One approach to address this latter problem is to develop individualized tumor vaccination to target mutated tumor surface proteins of individual patients. With the advent of advanced vaccination technologies, it is possible to generate individual vaccines to treat each patient according to the tumor’s individual mutational profile [19, 20]. This approach is difficult to achieve for intracellularly located proteins with tumor-specific mutations since antibodies mainly address extracellular rather than intracellular epitopes in living cells (although endocytic antibody internalization may take place). Therefore, therapeutic strategies are required to address intracellular tumor proteins, which constitute a considerable – if not the largest – portion of the tumor proteome.

In an endeavor to device new strategies for precision medicine based on small molecules to attack mutated intracellular proteins, we developed a concept, which integrates transcriptomic data with virtual drug screening of chemical libraries.

A central element in this context may be the concept of drug repurposing. Drug repurposing is a promising strategy and based on the successful usage of already approved drugs, which were initially developed for other indications than cancer [21]. One of the advantages of these drugs is that they already passed clinical safety testing in clinical trials. Considerable investments in terms of money and manpower are being spent to generate clinical evidence of safety and tolerability of a new drug to fulfill the high standards of the drug-approving authorities. Hence, drug repurposing is attractive from economic as well as medical points of view. The identification of thalidomide against severe erythema nodosum leprosum and retinoic acid against acute promyelocytic leukemia are two successful examples of drug repositioning [22, 23]. Plerixafor was initially developed as HIV drug to block viral entry into the cell, but clinical trials failed. However, leukocytosis in peripheral blood CD34 hematopoietic stem cells led to repurposing of plerixafor as stem cell mobilizing drug [24]. Our group has recently reported on drug repurposing of the anti-malarial artesunate for cancer therapy [25], e.g. prostate carcinoma [26] and colorectal cancer [27] as well as for other diseases such as viral infections [28] and schistosomiasis [29]. In addition, we found that the anti-fungal niclosamide exerted cytotoxic activity towards multidrug-resistant leukemia [30].

In the present study, we identified novel mutations in a glioblastoma patient and first screened a chemical library of more than 1500 FDA-approved drugs to find established drugs, which target novel BRAF and PIK3R1 mutations. Furthermore, we screened more than 25,000 novel compounds from the ZINC database. Then, the cytotoxicity of the selected compounds was evaluated. Furthermore, the protein expression of BRAF, PIK3R1 and other cancer biomarker proteins in the brain tumor tissue obtained from the patient was analyzed by immunohistochemistry. Finally, we identified novel small molecules that effectively targeted cells carrying mutant BRAF and PIK3R1, implying their potential for personalized glioblastoma therapy.

## Methods

### Patient characteristics

The patient characteristics were recently described [31]. In brief, a 65-year old patient had been diagnosed with glioblastoma WHO grade IV on May 26th, 2014. Informed written consent had been given by the patient that the data are allowed to be scientifically used and published (dated: January 26, 2016). The patient was enrolled in the INTRAGO trial [32], which was registered at clinictrials.gov, no. NCT02104882 (registration date: 03/26/2014) (https://clinicaltrials.gov/ct2/show/NCT02104882). INTRAGO was approved by the local ethics committee (Medical Ethics Commission II of the Faculty of Mannheim, University of Heidelberg 2013-548S-MA) and the Federal Office of Radiation Protection (Z 5-2246/2-2013-063). Temozolomide-based radiochemotherapy had been applied according to the current standard of care [32]. Treatment led to stable disease until May 2017, when surgery of the progressive tumor became necessary.

Transcriptome sequencing of tumor and normal tissue was performed at the National Center for Tumor Diseases (NCT, Heidelberg, Germany), which confirmed the histological diagnosis of glioblastoma. In total, 65 nucleotide substitutions, three focal and 17 larger copy number changes, as well as *MGMT* promoter methylation was found.

### Magnetic resonance imaging (MRI) and computer tomography (CT)

**Fig. 1:**
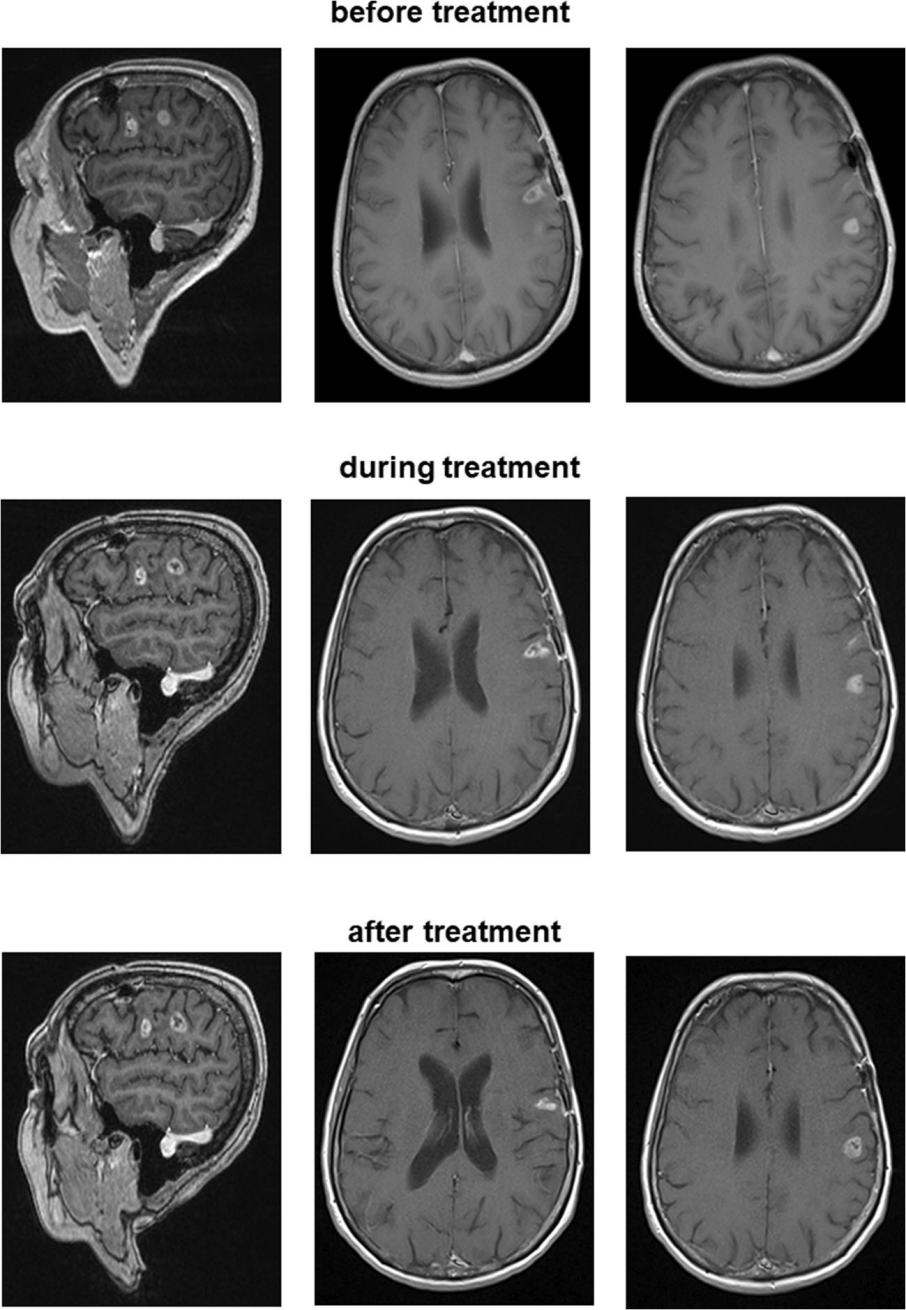
Computed tomography (CT) scans and magnetic resonance images (MRI) before, during and after the temozolomide treatment of the glioblastoma patient

Tumor development at the glioblastoma patient within 1 year (before, during and after temozolomide treatment) can be observed from the MRI scans depicted in Fig. 1. Tumor shrinkage occurred after temozolomide treatment.

### *In silico* analyses

A library of FDA-approved drugs (> 1,500 compounds) (http://zinc15.docking.org/substances/subsets/fda/) was screened for established drugs binding with high affinity towards wildtype BRAF, BRAF47-438del and PIK3R1G376R. The PyRx software [33] was used for initial virtual drug screening. The 10 top-ranked compounds with the highest affinities were selected for further analyses. As a second step, the ZINC database library [34] was used (> 25,000 compounds from the “all clean subset with pH 7”) to identify novel non-approved investigational compounds. Again, the 10 top-ranked compounds with the highest affinities towards BRAF47-438del and the 10 top-ranked compounds with the highest affinities towards PIK3R1G376R were selected for further analyses. The selected candidate compounds were further subjected to molecular docking with AutoDock 4 [35]. Whole protein surfaces were taken into account for virtual screening and molecular docking. Three independent docking calculations were conducted, with 25,000,000 energy evaluations and 250 runs by using the Lamarckian Genetic Algorithm. The corresponding lowest binding energies (LBE) were obtained from the docking log files (dlg), and mean values ± SD were calculated. For visualization of docking results, AutoDock Tools and Visual Molecular Dynamics (VMD) were used (Theoretical and Computational Biophysics group at the Beckman Institute, University of Illinois at Urbana-Champaign) (http://www.ks.uiuc.edu/Research/vmd/). Two known BRAF inhibitors (sorafenib [36] and vemurafenib [37]), two known PIK3R1 inhibitors (PI-103 [38] and LY-294,002 [39]) were used as control drugs.

### Immunohistochemistry

For immunohistochemical protein detection, we applied the UltraVision polymer detection method (kit from Thermo Fisher Scientific GmbH, Dreieich, Germany). The method was previously described in detail [26]. Briefly, formalin-fixed, and paraffin-embedded tumor tissue was incubated in a humidified chamber for 1 h at room temperature with primary antibodies against BRAF (1:100, polyclonal, Life Technologies GmbH, Darmstadt, Germany), PI3KR1 (1:100, clone U5, Life Technologies GmbH, Darmstadt, Germany), EGFR (1:50, clone H11, Life Technologies GmbH, Darmstadt, Germany), SRC (1:200, clone 1F11, Life Technologies GmbH, Darmstadt, Germany), AKT (1:100, clone 9Q7, Life Technologies GmbH, Darmstadt, Germany), mTOR (1:100, clone F11, Life Technologies Europe BV, Bleiswijk, Netherlands), NF-κB (1:100, polyclonal, Life Technologies GmbH, Darmstadt, Germany), or Ki-67 (ab16667, dilution 1:100, Abcam, Cambridge, UK). Afterwards, Primary Antibody Amplifier Quanto (Thermo Scientific) was applied for 10 min at room temperature, and HRP Polymer Quanto (Thermo Scientific) was applied for another 10 min. Protein expression was visualized by diaminobenzidine (DAB). Quanto chromogen (Thermo Scientific) was mixed with 1 ml DAB Quanto. The tissues were counterstained in hemalaun solution (Merck KGaA, Darmstadt, Germany). Standard histochemical staining was performed with hematoxylin-eosin (HE).

The immunostained slides were scanned by Panoramic Desk (3D Histotech Pannoramic digital slide scanner, Budapest, Ungary) and quantified by panoramic viewer software (NuclearQuant and MembraneQuant, 3D HISTECH) as previously described [26].

### NCI cell lines

The panel of cell lines of the Developmental Therapeutics Program (National Cancer Institute, USA) consists of 7 brain tumor cell lines (SF-295, SF-539, SNB-19, SNB-75, SNB-78, U251, XF498) and of cell lines from other tumor origins (leukemia, melanoma as well as carcinoma of the breast, ovary, prostate, lung, colon, and kidney). The origin of the cell lines has been described [40]. Up to 70 cell lines have been tested per drug using a sulforhodamine assay [41]. Some of these drugs identified by our virtual drug screening approach have been tested in this panel of cell lines, and the log_10_IC_50_ values have been deposited at the NCI website (https://dtp.cancer.gov/) [42].

### Cytotoxicity

Fifty per cent inhibitory concentrations (IC_50_) values for the selected compounds from virtual screening of FDA approved drugs towards the brain cancer cell line panel of the NCI cell lines were selected from the National Cancer Institute (NCI) database (http://dtp.nci.nih.gov). The cytotoxicity for the NCI cell line panel was assayed by the sulforhodamine B test, as previously described [43].

## Results

### Genotyping

**Fig. 2:**
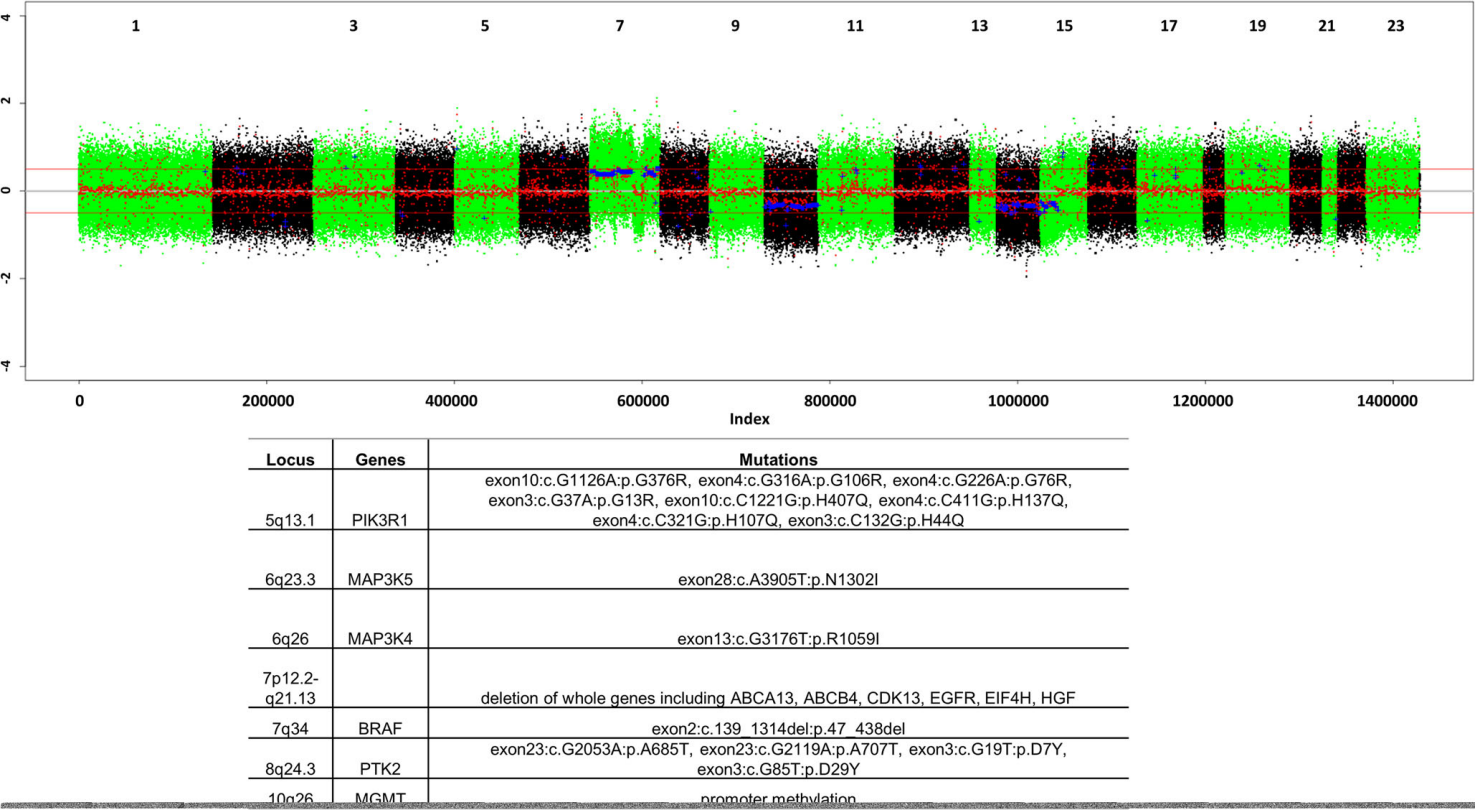
Copy number variation plot and the relevant mutations in the glioblastoma patient

The copy number variation plot with selected mutations and variations obtained from tumor genome sequencing of a glioblastoma patient is depicted in Fig. 2. Specific mutations have been identified in *BRAF* (BRAF47-438del, BRAF-TTYH3 fusion), *PIK3R1, MAP3K4, MAP3K5*, and *PTK2*. The promoter of *MGMT* was methylated. Additionally, several genes were completely deleted, *i.e. ABCA13, ABCB4, CDK13, EGFR, EIF4H*, and *HGF*).

### Immunohistochemistry

**Fig. 3:**
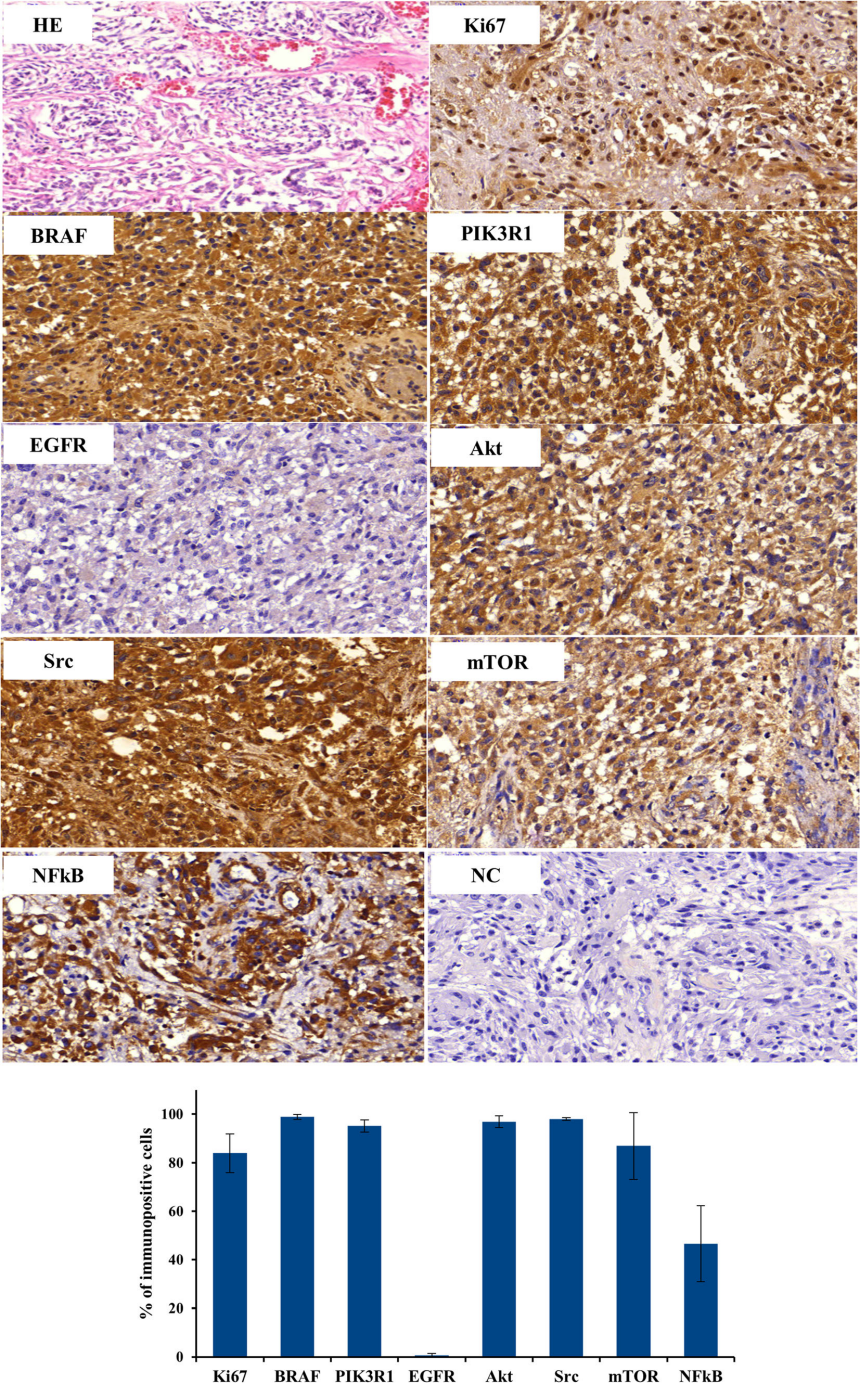
Immunohistochemical detection of BRAF, PIK3R1, EGFR, AKT, SRC, mTOR, NF-kB, Bar, 50 µM. HE, hematoxylin-eosin staining; NC, negative control (w/o primary antibody)

In order to confirm the relevance of the results of RNA-sequencing, we performed immunohistochemistry for selected markers, i.e. BRAF, PIK3R1, and EGFR. In addition, signaling molecules in the EGFR downstream signaling cascade have been investigated, e.g. kinases (AKT, SRC) and transcription factors (mTOR, NF-κB). Furthermore, general tumor features have been investigated by hematoxilin-eosin staining to monitor the typical glioblastoma multiforme morphology and by the immunohistochemical detection of Ki-67 to estimate the proliferation rate of the tumor. As shown in Fig. 3, all tumor markers except EGFR revealed strong immunopositivity. The genotyping data revealed EGFR whole gene deletion and this is confirmed by EGFR staining. The protein expression of BRAF and PIK3R1, as well as the lack of EGFR expression, fit well to the data obtained by RNA-sequencing. The expression of the additional markers (AKT, SRC, mTOR, NF-κB, Ki-67) are clues for the aggressiveness of the tumor.

### *In silico* analyses

In order to search for novel treatment options based on individual mutational profiles of glioblastoma genomes, we used the mutations of this patient for further bioinformatical analyses. We focused on the BRAF47-438del and PIK3R1G376R mutations because genetic alterations in these two genes are frequently assumed as driver mutations with relevance for tumor development and progression. The other mutations in the *MAP3K4*, *MSP3K5* and *PTK2* genes were not further considered.

The BRAF47-438del is a novel mutation found in this patient for the first time. Therefore, we compared this novel mutation with the most common BRAFV600E mutation. The BRAF-TTYH3 fusion protein could not be used for virtual drug screening, as no crystal structure of this protein was available in the Protein Data Bank (https://www.rcsb.org/). Therefore, a reliable homology model of this fusion protein could not be generated on the basis of the single wildtype BRAF and TTYH3 proteins.

**Fig. 4:**
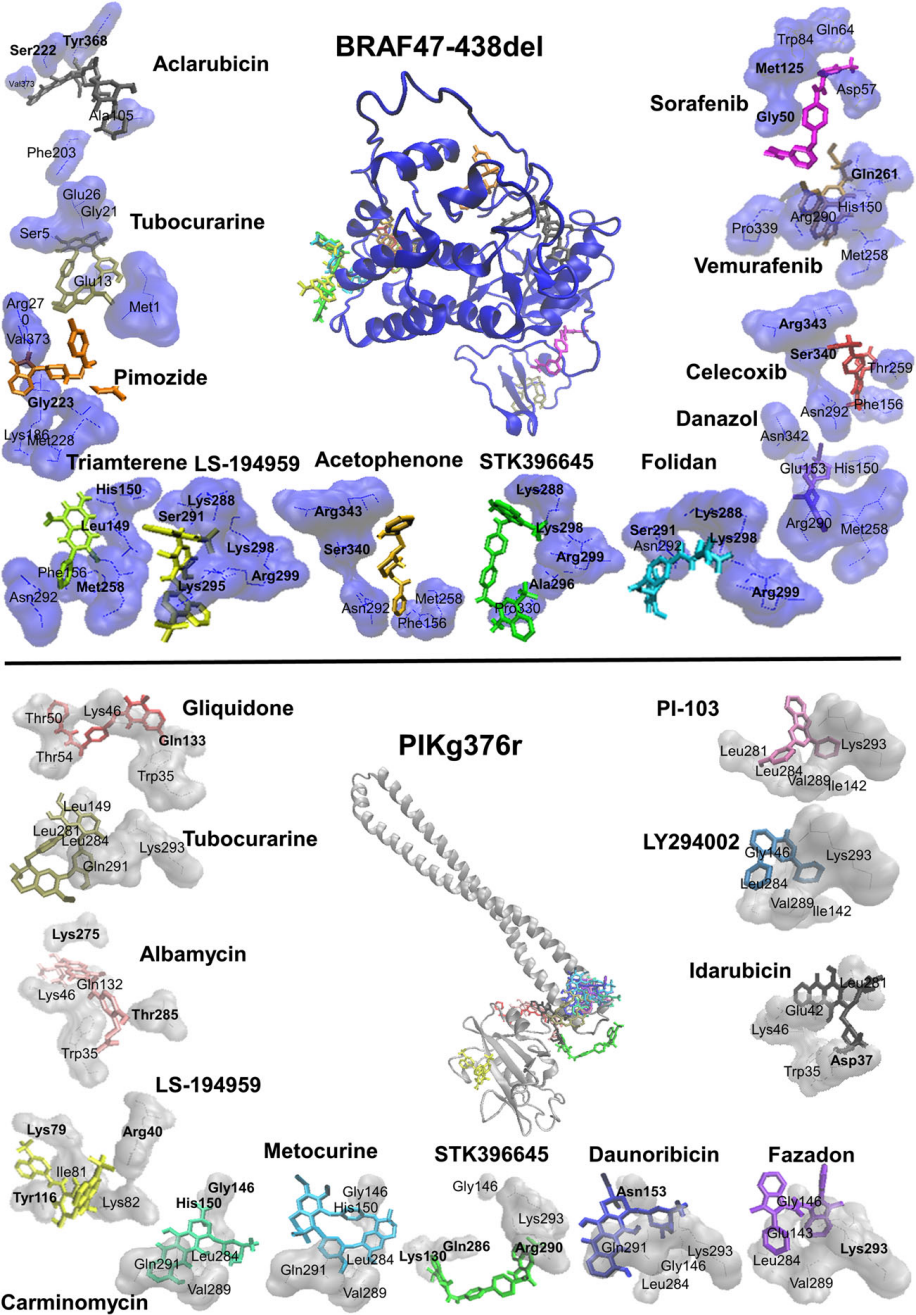
Docking poses of the top 10 ranked FDA-approved established drugs on the BRAF47-438del and PIK3R1G376R mutant proteins

Using PyRx and AutoDock 4.2, virtual screening of the library of FDA-approved drugs revealed drugs with high affinities towards BRAF47-438del and PIK3R1G376R (Table 1). The docking poses of top 10 compounds as well as of vemurafenib and sorafenib (control drugs for BRAF), LY-294,002 and PI-103 (control drugs for PIK3R1) are visualized in Fig. 4. With regard to the PyRx screening results, all 10 compounds revealed a stronger affinity to BRAF47-438del than vemurafenib and sorafenib. Concerning PIK3R1G376R, the top 10 compounds revealed stronger interaction than LY-294,002. All top 10 compounds except tubocurarine, metocurine and idarubicin possess higher affinity than PI-103. Concerning the BRAF47-438del AutoDock results, STK396645, pimozide, folidan, acetophenone, LS-194,959, danazol revealed stronger interaction than sorafenib. STK396645, pimozide, folidan, acetophenone, LS-194,959 also have stronger affinity than vemurafenib. Within the compounds revealed by PIK3R1G376R docking results, all top 10 compounds except albamycin, daunorubicin, tubocurarine had higher affinities than PI-103, whereas all compounds except albamycin, daunorubicin revealed stronger binding than LY-294,002. The established anti-BRAF drug sorafenib bound with slightly less affinity to BRAF47-438del (-9.39 ± 0.09 vs. -9.57 ± 0.22 kcal/mol) than to the corresponding wildtype protein as observed in molecular docking analyses. The established anti-PIK3R1 inhibitors; LY-294,002 and PI-103 revealed stronger binding to PIK3R1G376R mutant protein than wildtype protein (-6.47 ± < 0.01 vs. -5.19 ± 0.02 for LY-294,002 and − 7.22 ± 0.01 vs -5.76 ± 0.06 kcal/mol for PI-103). Accordingly, it was possible to identify drugs that bind stronger to BRAF47-438del than sorafenib and vemurafenib and drugs that stronger bind to PIK3R1G376R than LY-294,002 and PI-103, all of those can specifically target mutant proteins.

**Table 1:** Top 10 out of > 1500 FDA-approved established drugs with highest binding affinities towards mutant proteins (LBE = lowest binding energy, kcal/mol, pKi = predicted inhibition constant, µM)

	LBE			
BRAF47-438del	PyRx	AutoDock	pKi	Interacting residues
Tubocurarine	-11.0	-8.61 ± 0.01	0.48 ± < 0.01	Met1, Ala3, Ser5, Gly11, Ala12, Glu13, Gly15, Leu18, Gly21, Asp22, Glu26
STK396645	-10.9	-13.78 ± 0.45	0.0001 ± < 0.001	**Lys288, Ala296, Lys298, Arg299**, Lys291, Cys293, Pro294, Lys295, Leu329, Pro330
Celecoxib	-10.7	-8.94 ± 0.01	0.280 ± 0.004	**Ser340, Arg343**, Glu153, Phe156, Met158, Leu161, Thr259, Asn292, Glu338
Aclarubicin	-10.6	-8.39 ± 0.81	1.09 ± 0.88	**Ser222, Tyr368**, Ser75, Phe76, Ala105, Asn108, Asp184, Lys186, Phe203, Gly204, Leu205, Ala206, Thr207, Gln220, Ala370, Val373
Pimozide	-10.4	-10.55 ± 0.27	0.02 ± 0.01	**Gly223**, Lys186, Leu221, Ser222, Gly223, Ser224, Ile225, Met228, Leu262, Arg270, Ile363, Ala365, Ala370, Phe371, Val373, His374
Folidan	-10.3	-10.98 ± 0.30	0.009 ± 0.004	**Lys288, Ser291, Lys298, Arg299**, Asn292, Cys293, Pro294, Lys295, Arg334, Ser335
Acetophenone	-10.2	-10.48 ± 0.04	0.02 ± 0.02	**Ser340, Arg343**, Phe156, Glu157, Met158, Leu161, Met258, Gln261, Leu286, Asn292, Glu338, Pro339, Leu341
LS-194,959	-10.2	-13.34 ± 0.21	0.0002 ± < 0.001	**Lys288, Ser291, Lys295, Lys298, Arg299**, Cys293, Pro294, Ala296
Danazol	-10.2	-9.41 ± < 0.01	0.126 ± 0.0004	His150, Glu153, Met258, Thr259, Gln261, Arg290, Asn292, Glu338, Leu341, Asn342
Triamterene	-10.1	-7.69 ± 0.01	2.29 ± 0.01	**Leu149, His150, Met258**, Glu153, Phe156, Glu157, Met158, Leu161, Asn292, Glu338, Leu341
sorafenib	-8.5	-9.39 ± 0.09	0.13 ± 0.02	Leu49, **Gly50**, Arg51, Arg52, Asp57, Trp58, Ile60, Gln64, Trp84, **Met125**
vemurafenib	-7.8	-10.17 ± 0.16	0.04 ± 0.01	Leu149, His150, Glu153, Phe156, Met258, Thr259, Gly260, Gln261, Arg290, Asn292, Pro339, Ser340, Asn342, Arg343
PIK3R1G376R	PyRx	AutoDock	pKi	Interacting residues
LS-194,959	-9.9	-8.07 ± 0.05	1.22 ± 0.10	**Arg40, Lys79**, Leu80, Ile81, Lys82, **Tyr116**
STK396645	-9.7	-9.66 ± 0.16	0.08 ± 0.02	Asp37, **Lys130**, Ile142, Met282, Thr285, **Gln286**, Lys287, Gly288, Val289, **Arg290**
Carminomycin	-9.3	-7.36 ± 0.51	5.21 ± 4.75	Glu143, **Gly146**, Leu149, **His150**, Leu284, Thr285, Val289, Gln291
Albamycin	-9.3	-5.97 ± 0.07	42.39 ± 4.96	**Lys275, Thr285**, Trp35, Ile38, Glu42, Lys46, Val128, Gln132, Asp278, Gln279, Leu281, Met282,
Gliquidone	-9.2	-7.38 ± 0.59	5.56 ± 5.67	Trp35, Lys46, Thr50, Ala51, Thr54, Tyr126, Pro127, Val128, Lys130, Gln132, **Gln133**, Gln279, Met282
Fazadon	-9.2	-7.73 ± 0.06	2.15 ± 0.19	Glu143, Gly146, Leu149, Asn153, Leu284, Val289, **Lys293**
Daunorubicin	-9.1	-6.40 ± 0.67	27.83 ± 19.78	**Asn153**, Gly146, Leu149, Arg277, Leu281, Leu284, Thr285, Gln291, Lys293
Tubocurarine	-9.0	-6.72 ± 0.02	11.76 ± 0.38	Leu149, Glu42, Arg277, Leu281, Leu284, Thr285, Gln291, Lys293
Metocurine	-8.9	-7.30 ± 0.01	4.48 ± 0.03	Gly146, Leu149, His150, Asn153, Arg277, Leu281, Leu284, Val289, Gln291
Idarubicin	-8.8	-7.32 ± 0.76	7.61 ± 9.51	Trp35, **Asp37**, Glu42, Lys46, Leu281, Met282, Thr285
PI-103	-9.0	-7.22 ± 0.01	5.10 ± 0.08	Ile142, Gly146, His150, **Leu281**, Leu284, Val289, Gln291, Lys293
LY-294,002	-8.1	-6.47 ± < 0.01	18.07 ± 0.02	Ile142, Glu143, Gly146, Leu284, Val289, Lys293

**Fig. 5:**
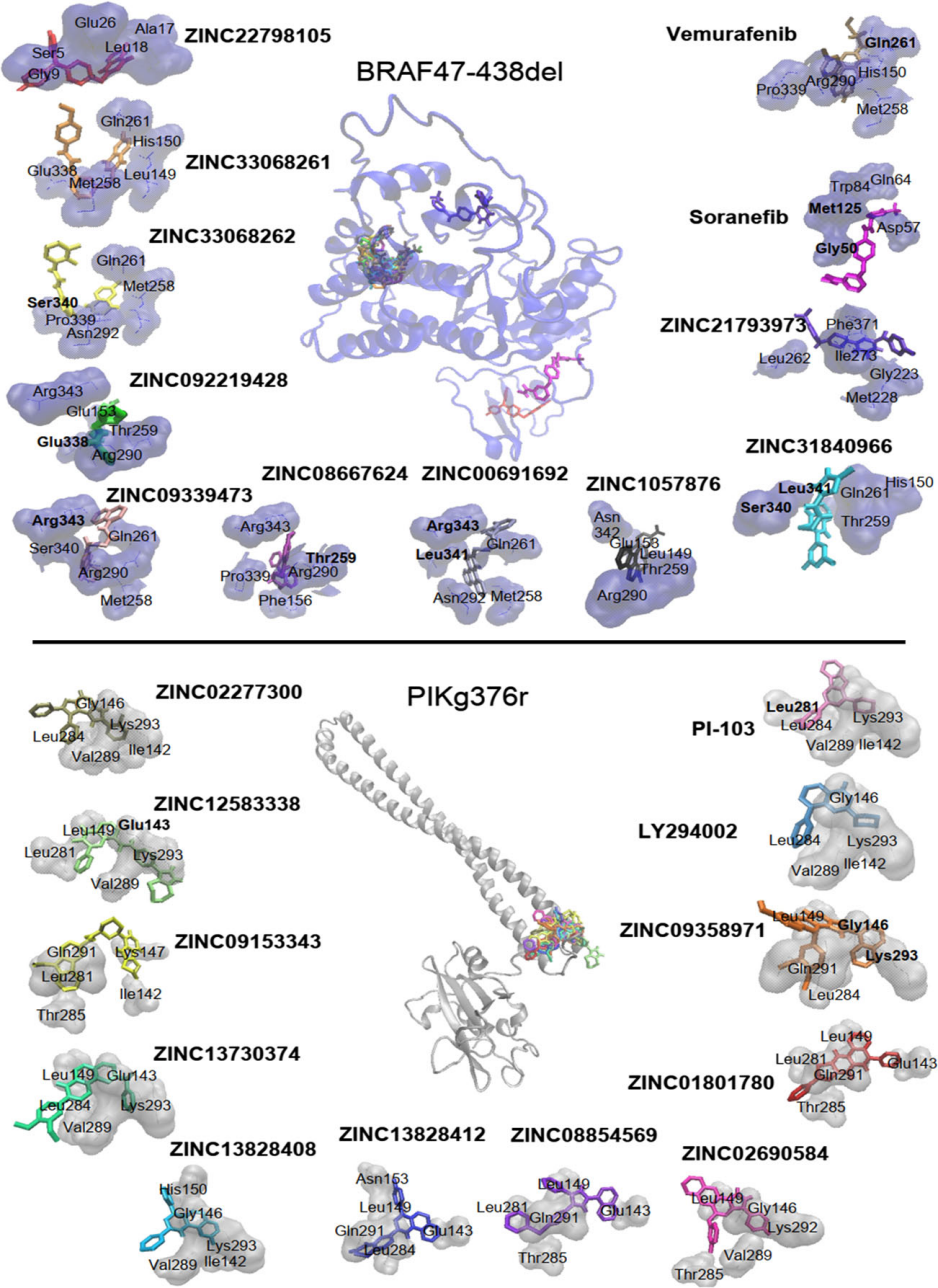
Docking poses of the top 10 ranked non-approved investigational compounds of the ZINC database to the BRAF47-438del and PIK3R1G376R mutant proteins

Furthermore, 10 investigational non-approved compounds from the ZINC database were identified for BRAF47-438del and PIK3R1G376R mutated proteins (Table 2). Residues forming hydrogen bonds were labeled in bold. Docking poses are depicted in Fig. 5. With regards to the PyRx results, all compounds revealed stronger interaction than the control compounds. BRAF47-438del AutoDock results pointed out that all compounds interact stronger than sorafenib. ZINC09339473, ZINC22798105, ZINC33068262, ZINC00691692, ZINC08667624, ZINC33068261, ZINC09219428, ZINC31840966 interact stronger than both vemurafenib and sorafenib. PIK3R1G376R AutoDock results revealed that all compounds except ZINC02690584 interact stronger than both PI-103 and LY-294,002 (Table 3).

**Table 2:** Top 10 out of > 25,000 non-approved investigational compounds from the ZINC database with highest binding affinities towards mutant proteins (LBE = lowest binding energy, kcal/mol, pKi = predicted inhibition constant, µM)

	LBE			
BRAF47-438del	PyRx	AutoDock	pKi	Interacting residues
ZINC09339473	-12.3	-11.30 ± 0.28	0.006 ± 0.003	Phe156, Met258, Thr259, Gln261, Ser265, Leu286, Arg290, Asn292, Glu338, Ser340, Asn342, **Arg343**, Ala344
ZINC10578766	-12.3	-10.16 ± 0.47	0.043 ± 0.033	Leu149, His150, Glu153, Thr154, Met258, Thr259, Gln261, Arg290, Glu338, Ser340, Leu341, Asn342
ZINC22798105	-12.2	-10.32 ± 0.24	0.03 ± 0.01	Ser5, Gly6, Gly9, Ala12, Gly15, Ala17, Leu18, Gly21, Asp22, Glu24, **Glu26**
ZINC33068262	-11.8	-11.39 ± 0.36	0.005 ± 0.003	Leu149, Met258, Thr259, Gln261, Leu286, Val289, Arg290, Asn292, Glu338, Pro339, **Ser340**, Arg343, Ala344
ZINC00691692	-11.8	-10.87 ± 0.21	0.014 ± 0.006	Leu161, Met258, Thr259, Gln261, Ser265, Leu286, Arg290, Asn292, Cys293, Glu338, Ser340, **Leu341, Arg343**
ZINC08667624	-11.8	-12.35 ± 0.23	0.001 ± < 0.001	Glu153, Phe156, Leu161, Met258, **Thr259**, Gln261, Arg290, Asn292, Glu338, Pro339, Ser340, Leu341, Arg343
ZINC33068261	-11.7	-11.41 ± 0.10	0.004 ± 0.001	Leu149, His150, Glu153, Phe156, Leu161, Met258, Thr259, Gly260, Gln261, Arg290, **Glu338**, Pro339, Ser340
ZINC09219428	-11.7	-10.19 ± 0.39	0.039 ± 0.027	His150, Glu153, Leu161, Met258, Thr259, Gln261, Arg290, Asn292, Cys293, **Glu338**, Ser340, Leu341, Asn342, Arg343
ZINC21793973	-11.6	-9.66 ± 0.01	0.083 ± 0.002	Ser222, Gly223, Ile225, Leu226, Met228, Ile233, Leu262, Ile267, Arg270, Ile273, Ile363, Ala370, Phe371, Val373
ZINC31840966	-11.6	-10.81 ± 0.17	0.012 ± 0.004	Leu149, His150, Met158, Leu161, Met258, Thr259, Gln261, Asn292, Cys293, Glu338, **Ser340, Leu341**, Asn 342, Arg343, Ala344
sorafenib	-8.5	-9.39 ± 0.09	0.13 ± 0.02	Leu49, **Gly50**, Arg51, Arg52, Asp57, Trp58, Ile60, Gln64, Trp84, **Met125**
vemurafenib	-7.8	-10.17 ± 0.16	0.04 ± 0.01	Leu149, His150, Glu153, Phe156, Met258, Thr259, Gly260, Gln261, Arg290, Asn292, Pro339, Ser340, Asn342, Arg343
PIK3R1G376R	PyRx	AutoDock	pKi	Interacting residues
ZINC12583338	-10.8	-8.73 ± 0.24	0.42 ± 0.15	Ile142, **Glu143**, Gly146, Leu149, Leu281, Leu284, Val289, Gln291, Lys293
ZINC13828412	-10.3	9.27 ± < 0.01	0.16 ± < 0.01	Ile142, Glu143, Gly146, Leu149, His150, Asn153, Leu281, Leu284, Thr285, Val289, Arg290, Gln291
ZINC08854569	-10.1	-8.08 ± 0.20	1.25 ± 0.45	Ile142, Glu143, Gly146, Leu149, Leu281, Leu284, Thr285, Gln291
ZINC01801780	-10	-7.36 ± 0.01	4.00 ± 0.01	Glu143, Glu146, Leu149, Leu281, Leu284, Thr285, Val289, Gln291
ZINC02277300	-10	-7.66 ± 0.01	2.41 ± 0.02	Ile142, Glu143, Gly146, Leu281, Leu284, Val289, Gln291, Lys293
ZINC09358971	-9.9	-7.85 ± 0.04	1.77 ± 0.12	Glu143, **Gly146**, Leu149, Leu284, Thr285, Val289, Gln291, **Lys293**
ZINC02690584	-9.8	-6.73 ± 0.01	11.59 ± 0.22	Gly146, Leu149, Leu284, Thr285, Val289, Lys292
ZINC09153343	-9.8	-8.04 ± 0.12	1.29 ± 0.27	Ile142, Glu143, Gly146, Lys147, Leu149, Leu281, Leu284, Thr285, Val289, Gln291, Lys293
ZINC13730374	-9.8	-9.02 ± 0.01	0.24 ± < 0.01	Ile142, Glu143, Gly146, Leu149, Leu281, Leu284, Thr285, Val289, Gln291, Lys293
ZINC13828408	-9.8	-7.87 ± 0.01	1.69 ± 0.02	Ile142, Glu143, Gly146, Leu149, His150, Leu284, Val289, Gln291, Lys293
PI-103	-9.0	-7.22 ± 0.01	5.10 ± 0.08	Ile142, Gly146, His150, **Leu281**, Leu284, Val289, Gln291, Lys293
LY-294,002	-8.1	-6.47 ± < 0.01	18.07 ± 0.02	Ile142, Glu143, Gly146, Leu284, Val289, Lys293

**Table 3:** Disease indications and modes of action of FDA-approved drugs identified by virtual drug screening

Drug	Disease/application	Mode of action	Origin	Potentially recommendable for therapy
BRAF47-438del:				
Tubocurarine	Arrow poison (main component of curare)	Non-depolarizing muscle relaxant; competitive inhibitor of acetylcholine receptors at the postsynaptic membrane; inhibition of ligand-driven natrium ion channels.	*Condrodendron tomentosum*	No
STK396645				No
Celecoxib	Degenerative joint diseases (arthrosis), rheumatoid arthritis; Morbus Bechterew (Spondylitis ankylosans)	Non-steroidal anti-rheumatic; selective COX2 inhibitor; inhibition of prostaglandin synthesis	Synthetic	Yes
Aclarubicin	Cancer	Anthracycline; histone eviction from chromatin upon DNA intercalation	*Strepomyces galilaeus*	Yes
Pimozide	Chronic schizophrenic psychoses	Antipsychotic; postsynaptic inhibition of dopamin receptors and thereby stimulation of presynaptic dopamin release; inhibition of acidic sphingomyelinase	Synthetic	Yes
Folidan (Calcium folinate, Leucovorin)	Thrombocytopenia, neutropenia, anemia;	Folic acid source to prevent depletion by folic acid antagonists; counteracts toxicity by folic acid antagonists	Synthetic	Yes
Acetophenone	chemical precursor for the synthesis of flagnaces and pharmaceuticals	Hypnotic, anticonvulsant, sedative	Ingredient of essential oils (labdanum, castoreum, *Stirlingia latifolia*)	No
LS-194,959	Cancer	Inhibitor of CDK2	synthetic	No
Danazol	Endometriosis	Testosterone derivative; inhibitor of gonatropine release (FSH, LH); interruption of telomere erosion	Semisynthetic	Yes
Triamterene	Hypertonia; chronic heart insufficiency	Natrium-sparing diuretic; inhibition of aldosteron-dependent epithelial natrium channels in late distal tubuli	Synthetic	Yes
PIK3R1G376R:				
LS-194,959	see above			No
STK396645	see above			No
Carminomycin	Cancer	Anthracycline; DNA intercalation; DNA topoisomerase II inhibitor	*Actinomadura carminata*	Yes
Novobiocin	Bacerial infections	Aminocoumarin antibiotic; inhibition of bacterial gyrase, subunit GyrB	*Streptomyces niveus*	Yes
Gliquidone	Diabetes mellitus	Sulyonylurea; inhibition of natrium channels and depolarization of B-cells leading to opening of current-regulated potassium channels. The potassium influx depletes insulin from storage vesicles and increases insulin release into the blood.	Synthetic	Yes
Fazadon (fazadinium bromide)	Anaesthetic in otorhinolaryngological surgery	Non-depolarizing muscle relaxant; antagonist of acetylcholine through neuromuscular blockage	Synthetic	Yes
Daunorubicin	Acute leukemia	Anthracycline; DNA intercalaction; DNA topoisomerase II inhibitor; generation of cytotoxic superoxide. And hydroxyl-radicals	*Streptomyces peucetius* and *S. coeruleorubidus*	Yes
Tubocurare	see above			
Metocurine	Convulsive conditions: anesthetic adjuvant	Non-depolarizing muscle relaxant; antagonist of acetylcholine by competitive binding to cholinergic receptors at the motor-end plate	Synthetic	Yes
Idarubicin	Acute leukemia	Anthracycline; DNA intercalaction; DNA topoisomerase II inhibitor	Semisynthetic	Yes

### Cytotoxicity

**Fig. 6:**
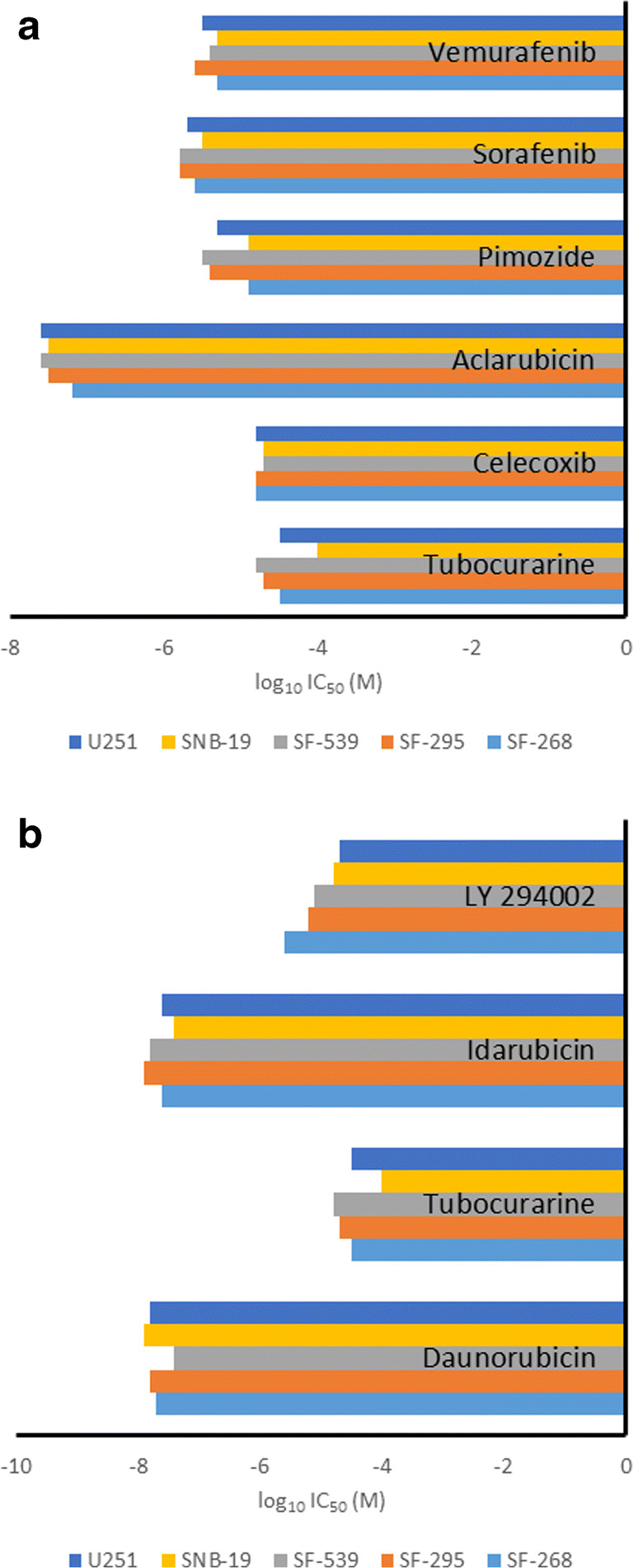
Cytotoxicity of the available top 10 ranked FDA-approved established drugs (**a** BRAF-47-438del screening, **b** PIK3R1-G376R screening) and the known inhibitors towards brain cancer cell lines of the NCI (Bethesda, USA) panel. Shown are log_10_IC_50_ values (M)

Virtual drug screening revealed that anthracyclines, among other drugs, might target the mutated BRAF and PIK3R1 proteins. Since these drugs are not clinically used for the treatment of glioblastoma multiforme, we explored whether these compounds are able to kill glioblastoma cells *in vitro* with comparable efficacy than cell lines from other tumor origin. For this reason, we screened the NCI database for test results of the compounds by our virtual drug screening approach. The log_10_IC_50_ values of several brain tumor cell lines for the corresponding compounds included into this database are shown in Fig. 6. For comparison, the log_10_IC_50_ mean values for each of the test compounds of 46–63 other tumor cell lines have been calculated. The following observations were made: (1) the anthracyclines (daunorubicin, idarubicin, aclacinomycin) were the most cytotoxic compounds in this panel of drugs; (2) Pimazole also showed considerable cytotoxicity against tumor cells, although this is a psychoactive drug used for the treatment of chronic schizophrenic psychoses; (3) The cytotoxicity of the compounds tested were not significantly different between glioblastoma cell lines and all other tumor cell lines, indicating that these compounds might be effective in glioblastoma patients, given that appropriate formulations will be chosen to cross the blood-brain barrier. Aclarubicin revealed cytotoxicity (log_10_IC_50_) in the range of -7.2 to -7.6, higher than both sorafenib (in the range of -5.6 to -5.8) and vemurafenib (in the range of -5.3 to -5.6) (Fig. 6a). Idarubicin and daunorubicin (in the range of -7.4 to -7.9) revealed higher cytotoxicity than LY-294,002 (in the range of -4.7 to -5.6) (Fig. 6b).

## Discussion

Since many years, MRI/CT imaging is routinely used for monitoring treatment success. However, imaging cannot deliver hints, which salvage therapy could be applied if standard therapy fails. A novel concept in precision medicine is to sequence tumor transcriptomes to identify patient-specific mutations, which can be then addressed by targeted therapeutics [44].

In the present investigation, we explored the possibility to use transcriptomic information for virtual drug screening, in order to indicate novel treatment options for individual patients. For this purpose, we used the mutational profile of a glioblastoma patient as an example to prove the feasibility of this approach. We first focused on screening of FDA-approved drugs, since repurposing of drugs initially approved for other diseases than cancer provided interesting treatment alternatives in many cases. The mutation profile obtained by genotyping of the patient’s tumor has several implications for therapy:

1. The BRAF inhibitors vemurafenib and sorafenib may be less efficient to inhibit mutated BRAF proteins than wildtype BRAF.
2. Promoter-methylation of the *MGMT* gene indicates that MGMT protein expression may be downregulated. As MGMT represents a resistance factor for temozolomide [45], this drug may be exquisitely efficient to treat this patient. Indeed, the treatment history of the patient demonstrated a very good response to the temozolomide-based radiochemotherapy protocol.
3. The *EGFR* deletion indicated that EGFR-directed drugs (such as erlotinib, gefitinib and others) might not be effective.
4. Small molecules addressing the other mutations are not available yet, which limits the therapeutic options in this patient.
5. The *HGF* gene deletion might be relevant for toxic side effects. HGF (hepatocyte growth factor) is known to protect from drug-induced hepatotoxicity [46–48]. In fact, this patient experienced hepatotoxicity by radiochemotherapy with temozolomide and compassionate use of artesunate and Chinese herbs [31]. It can be speculated that the observed hepatotoxicity might be explained at least in part by the *HGF* deletion.

To validate the RNA-sequencing data, we performed immunohistochemical analyses. The strong immunostaining of BRAF and PI3KR1 indicated that the primary antibodies used for immunohistochemistry detect not only wildtype but also mutated forms of these proteins and that both proteins were expressed at high levels in this glioblastoma case. Accordingly, immuno-positive staining of tissue slices by these antibodies is probably not the suitable indicator for the usage of the compounds newly identified, whereas sequencing of tissue is more appropriate for the detection of the new mutations.

Furthermore, the sequencing results showed that the *EGFR* gene was deleted, which was confirmed by immunohistochemistry since the tumor was EGFR-negative in immunohistochemical staining. Even if EGFR is not expressed in this tumor, the question arises, whether downstream signaling pathways are still operative, which might then not be linked to EGFR but to other signaling molecules. We observed strong expressions of signaling kinases (SRC, AKT) and transcription factors (mTOR, NF-κB). Hence, these signaling molecules may still be susceptible to specific small molecule inhibitors against SRC, AKT, mTOR, or NF-κB) [49–52].

A limitation of the tumor sequencing approach is that therapeutic antibodies and small molecules are approved only for some of the major tumor-related proteins, but the vast majority of mutations cannot be therapeutically addressed as of yet. A premise to exploit the full potential of precision medicine would be that the therapeutic options keep pace with the diagnostic power and that ideally hundreds of drugs are available to inhibit the numerous tumor-related mutated proteins. An approach to reach this goal may be for instance tumor vaccination based on mutanomic profiling [20]. Comparable approaches are difficult to realize for small molecules, because the clinical approval of novel drugs is laborious and cost-intensive, and it can take years to bring a new drug to the market.

Therefore, it may be feasible to search for drugs, which have been already approved for other diseases and conditions and which also show activity against cancer by inhibiting tumor-related proteins. This approach has been termed drug repurposing and recently led to astonishing treatment successes [53–55].

In the present investigation, we attempted to utilize the wealth of information of RNA-sequencing data obtained from tumor genotyping to identify already FDA-approved drugs that bound with high affinity to mutated proteins in a glioblastoma patient. A database of > 1,500 FDA-approved drugs was first screened by PyRx. The results obtained were then refined by molecular docking with AutoDock 4.2. Furthermore, we used the novel BRAF47-438del mutation and the known PIK3R1G376R mutation as models to prove the feasibility of this approach. It is reasonable to search for drugs that stronger bind to BRAF47-438del than sorafenib and vemurafenib and to search for drugs that stronger bind to PIK3R1G376R than LY-294,002 and PI-103 to identify compounds that can specifically target mutant proteins. The BRAF47-438del docking results pointed out that STK396645, pimozide, folidan, acetophenone, LS-194,959, danazol bound stronger than sorafenib. STK396645, pimozide, folidan, acetophenone, LS-194,959 also had stronger affinities than vemurafenib. Considering the PIK3R1G376R results, all compounds except albamycin, daunorubicin, tubocurarine had higher affinities than PI-103, whereas all compounds except albamycin, daunorubicin revealed stronger binding than LY-294,002.

Virtual drug screening unraveled a panel of drugs that bound with high affinity to BRAF47-438del or PI3KR1G376R. These drugs were from diverse chemical classes and had different pharmacological activities. Strong poisons such as tubocurarine appeared in the screening, which cannot be considered for treatment. Remarkably, anthracyclines were also among the top-ranking drugs, which are known for their strong anticancer activity (daunorubicin, idarubicin, aclarubicin, carminomycin). Further drugs identified by virtual drug screening revealed anti-inflammatory (celecoxib), psycho-active (pimozide, acetophenone), muscle relaxant (fazadon, metocurine), diuretic (triamterene) antidiabetic (gliquidone), antibiotic (novobiocin), anti-endometriotic (danazol) or other pharmacological activities. The wide variety of drugs can be taken as a clue for the potential of repurposing drugs with diverse pharmacological modes of action for cancer therapy. The question arises, which of these drugs may be useful to treat the glioblastoma of the patient presented in our study. The decision may depend not only on the known side effects of these drugs but also on whether they are able to cross the blood-brain barrier (BBB). While this is well known for psychoactive drugs, anthracyclines are known substrates of the ATP-binding cassette (ABC) transporter, P-glycoprotein, which is an important constituent of the BBB. Anthracyclines such as daunorubicin, idarubicin and others are usually not used for the treatment of glioblastoma, since sufficient amounts cannot cross the BBB to exert considerable therapeutic anticancer effects in the brain [56]. This problem may, however, be tackled by novel nanotechnologically engineered anthracycline-releasing devices to overcome the BBB [57–59]. Since several anthracyclines appeared as top-ranked drugs to bind with high affinity to BRAF47-438del and PI3KR1G376R, it is worth reconsidering them for glioblastoma therapy, if appropriate nanotechnological formulations allow BBB-crossing. This problem may, however, be overcome, if appropriate nanotechnological formulations will be applied. There are daunorubicin liposomes preparations available on the market [60], and anthracycline liposomes have been shown to cross the blood-brain barrier [61–63].

Of course, anthracyclines cannot be viewed as mono-specific drugs addressing the two mutations in BRAF and PI3KR1 in an exclusive manner. As classical drugs of natural origin, they can be reconsidered to act in a multi-specific fashion. Anthracyclines are known to intercalate into DNA, to inhibit DNA topoisomerase II, and to generate cytotoxic superoxide- and hydroxyl radicals. Several other on- and off-target effects can be assumed, and binding to mutated BRAF and PI3KR1 proteins may belong to these still unknown modes of action of anthracyclines.

Having the multi-specific nature of many drugs in mind, it is reasonable to investigate their cytotoxic potential in cell lines with diverse mutations. Therefore, we compared the cytotoxicity of the drugs identified by our virtual drug screening approach in the panel of brain tumor cell lines of the Developmental Therapeutics Program of the National Cancer Institute (NCI, USA) and compared their activity with those of cell lines from other tumor origins. These results may deliver additional information, whether these drugs - initially developed for the treatment of other diseases - are suitable for drug repurposing in glioblastoma therapy. The data obtained with the NCI panel of cell lines pointed out aclarubicin, daunorubicin and idarubicin for glioblastoma treatment since they revealed higher cytotoxicity than the known inhibitors (sorafenib, vemurafenib and LY-294,002). Although the standard drug for glioblastoma treatment is temozolomide, anthracyclines also revealed strong cytotoxicity *in vitro* against brain tumor cell lines.

Pimozide is a psychoactive drug that does cross the BBB. Since this drug also revealed cytotoxicity towards brain tumor cell lines *in vitro*, its repurposing for clinical glioblastoma treatment may also be considered.

At the time point, when we obtained these results, the glioblastoma was already at a progressive state and the patient decided not to undergo any further drug treatment anymore. Unfortunately, the patient died before we could recommend an individualized treatment with a liposome-based anthracycline. Hence, a final proof of the clinical success of the concept presented in his paper could not be given. Nevertheless, our study represents a preclinical feasibility approach and a method to identify possible treatment candidates in cases that lack therapeutic options, which is a scenario frequently occurring in clinical practice.

We suggest that further investigations using the tumor genomes of patients should be made to predict active drugs and to observe whether treatment of patients with these drugs really leads to clinically significant response rates.

In general, repurposing of already approved drugs may not deliver more drugs for the individual treatment of cancer patients due to the multiplicity of tumor-related mutations. Therefore, we applied a second approach and used virtual drug screening to screen a chemical library of > 25,000 non-approved investigational compounds. We analyzed whether novel compounds can be identified that also bind to the BRAF and PI3KR1 mutations with high affinities. Indeed, most of the selected compounds revealed stronger binding than the known inhibitors. For instance, all of the selected compounds bound stronger to BRAF47-438del mutant protein than sorafenib and selected compounds except ZINC21793973 bound stronger than vemurafenib. The identified substances may be used as a starting point for further drug development to improve glioblastoma therapy in the future.

Despite the attractiveness of the virtual drug screening approach based on tumor sequencing data, there are also some limitations of this approach that have to be discussed:

1. Virtual drug screening of both FDA-approved drug as well as non-approved investigational compounds may result in the identification of a certain rate of false positive candidates [64, 65]. Therefore, results from virtual drug screening should be verified by *in vitro* or *in vivo* experiments.
2. The sequencing of tumor genomes and transcriptomes delivers information on both relevant driver as well as irrelevant passenger mutations regarding tumor development and progression. Hence, careful visual inspection of the sequencing data is advisable to make rational decisions, which mutated proteins are most suitable for virtual drug screening to find suitable candidate drugs with the therapeutic potential to significantly and sustainably improve tumor response to treatment and to prolong the survival time of patients.

## Conclusions

Systematic genome-dependent repurposing may reveal putative drug candidates for the further development of precision medicine in cancer and other gene-dependent diseases. A combined concept consisting of multiple techniques, i.e. tumor transcriptomics, diverse virtual drug screening methods, chemical libraries for drug repurposing and *in vitro* testing may help to make beneficial predictions on small molecules to inhibit distinct mutated proteins and to improve cancer therapy for each individual patient. We term the concept presented here “ReSeqtion”  (Repurposing of drugs by genome Sequencing and bioinformatic calculation).

## Data Availability

The data are available upon reasonable request.

## Abbreviations

AKT: AKT serine/threonine kinase 1
BBB: blood brain barrier
BRAF: B-Raf proto-oncogene, serine/threonine kinase
CD34: cluster of differentiation marker 34
CRISPR/Cas9: Clustered regularly interspaced short palindromic repeats/CRISPR-associated 3
CT: computer tomography
EGFR: epidermal growth factor receptor
FDA: Food and Drug Administration
HGF: hepatocyte growth factor
LBE: lowest binding energy
MAP3K4/5: Mitogen-activated protein kinase kinase kinase 4/5
MGMT: O-6-methylguanine-DNA methyltransferase
MRI: magnetic resonance imaging
NCI: National Cancer Institute
NF-κB: nuclear factor-kappa B
PIK3R1: Phosphoinositide-3-kinase regulatory subunit 1
PTK2: Protein tyrosine kinase 2
RMSD: root mean square deviation
SRC: SRC proto-oncogene, non-receptor tyrosine kinase
TTYH1: Tweety family member 1
VEGFR: vascular endothelial growth factor receptor
VMD: Visual Molecular Dynamics
WHO: World Health Organization
